# B-H Curve Estimation and Air Gap Optimization for High-Performance Split Core

**DOI:** 10.3390/ma18030644

**Published:** 2025-01-31

**Authors:** Minjoong Kim, Myungseo Lee, Sijeong Lee, Jaeyun Lee, Jihwan Song

**Affiliations:** 1 Department of Mechanical Engineering, Hanbat National University, 125 Dongseodae-ro, Yuseong-gu, Daejeon 34158, Republic of Korea; kimminjoong.mmm@gmail.com (M.K.); myungseo.mmm@gmail.com (M.L.);; 2 Corechips, 33 Omokcheon-ro Gwonseon-gu, Suwon 16642, Republic of Korea

**Keywords:** current transformer, B-H curve estimation, energy harvesting, air gap optimization, magnetic core design

## Abstract

The current transformer (CT)-based energy harvesting method has gained considerable attention for low-power devices. Accurate estimation of the B-H curve is essential to develop a high-performance CT, as it closely relates to the electromagnetic behavior of CT material. However, the existing estimation methods for the B-H curve face several drawbacks, which include process complexity and a high cost. This study presented an intuitive method to estimate the B-H curve based on the experimentally obtained resistance-voltage data. The performance of the CT core is obtained based on the estimated B-H curve, which exhibited an error of only 2.6% when compared to the experimental results for the most accurate case. Additionally, we analyzed split-core performance deterioration caused by the presence of an air gap. The air gap formation of the split core was closely related to the surface roughness, which significantly influenced core performance. The air gap range that minimizes the reduction in performance is predicted and validated through simulations and experiments. This research highlights a straightforward approach to obtaining the B-H curve of magnetic CT core material. We believe that this study provides the design guidelines needed to develop a high-performance CT core, including considerations for core geometry and the recommended air gap range.

## 1. Introduction

Energy harvesting is the process of storing and using energy for electrical devices by converting it from natural or external sources. In particular, energy harvesting has gained considerable attention in the field of wireless sensing as a method of supplying sustainable power to devices. Wireless sensing devices are used in structures such as substations, power lines, and buildings to monitor conditions such as temperature [1,2,3,4,5], structural deformation [4,5], and electric loads [3]. The commonly used energy harvesting methods include energy sources such as solar [6,7,8,9], wind [10,11,12], vibration [13], and magnetic fields [14,15]. Solar and wind generators face difficulties in supplying sustainable power since they depend considerably on weather conditions. Additional energy storage devices are required to overcome this problem. The vibration energy harvesters are unaffected by weather conditions; however, the generated power density decreases when certain conditions (e.g., resonant frequency) are not satisfied. A current transformer (CT) generates power by changing the magnetic field induced by an AC flowing through the power line. Therefore, the weather conditions do not affect the CT, and a high density of harvested energy can be obtained because this method does not require specific conditions to produce energy. The research on CT has been actively performed, considering their performance-enhancing advantages [2,3,4,5,14,15,16,17,18,19,20,21,22,23,24,25,26,27,28,29,30,31,32]. Consequently, the CT-based energy harvesting method is widely applied to low-power devices, such as maintenance [33,34] and lighting equipment [35] around power lines.

CTs play an essential role as electrical power sources in various fields; thus, it is important to understand the magnetic behavior of materials when designing high-performance CTs. Therefore, it is crucial to accurately estimate the B-H curve, which closely corresponds to the magnetic behavior of the CT material. In the early stages, the B-H curve was approximated using simple mathematical functions, such as the power series and exponential and hyperbolic functions [36,37,38,39,40]. However, such single-fitting functions cannot effectively approximate the entire region of the B-H curve. Especially, exponential function tends to produce significant errors near the origin and saturation point and the power series function struggles to reflect the trend in the saturation region, resulting in inaccurate approximations beyond this region. Thus, the B-H curve was divided into a spline curve by applying suitable functions to each section [41]. Additionally, an attempt was made to evaluate the B-H curve using a fuzzy model [42]. An accurate B-H curve was obtained by improving the experimental equipment, such as the vibration sample magnetometer (VSM) [43,44] and Epstein [43,45]. Unfortunately, VSM equipment is expensive and requires additional control units to maintain the experimental conditions, such as the temperature, during operation. In the case of Epstein, it is reliable only within a specific range of magnetic flux density due to the capacity of magnetic field generation. Thus, the region accompanies straight-line extrapolations (SLE) by necessity [46,47] and cannot consider magnetic saturation. Additionally, experimental methods face limitations in sample preparation. VSM requires the sample to weigh less than 10 g, and the Epstein requires four precisely prepared samples measuring 280 mm × 30 mm assembled into frame.

On the other hand, improving the performance of a CT requires the accurate estimation of the B-H curve of the CT materials as well as the effective core design of the CT. Split cores present the advantage of being easier to coil on the core and more convenient to connect power lines than ring cores [48]. Additionally, when the existing ring core-based CT exceeds the CT-rated current with the increase in the voltage level, short-circuit incidents and operation failures are observed because it is difficult to effectively transfer the primary current to the core [26,49]. Consequently, split cores are used more often than ring cores; however, they inevitably have air gaps that are closely related to the deterioration of the CT performance [50,51]. Several studies have been conducted, focusing on comparing their performance with ring cores, but only a few studies have been conducted on the performance with an air gap distribution in the split cores.

Herein, we propose an intuitive method to estimate the B-H curve directly from the experimentally measured resistance-voltage data using the actual CT core. In the proposed method, a large amount of data, collected under various current and resistance conditions, is leveraged, enabling the derivation of the B-H curve in the form of a trend line. The performance of the CT core through the obtained B-H curve exhibited high accuracy when compared to the experimental analysis. Additionally, geometric analysis was performed for the design of a high-performance CT core, and the performance of the split core was analyzed in the presence of an air gap, and the air gap range was also analyzed to ensure that the split core presents the expected performance. Interestingly, from the perspective of core design, it was confirmed that not only the volume of the core but also the ratio of its inner and outer radii are crucial parameters in determining its performance, and the split core has a similar performance to the ring core when the air gap is maintained within a specific range. We believe that the proposed intuitive estimation of the B-H curve and the analysis of the CT core performance can provide a useful guide for the design of a high-performance split core.

## 2. Materials and Methods

### 2.1. Experiment

The load current tester, oscilloscope, current probe, and load resistance are used to analyze the performance of the CT core, such as the induced voltage and the power for each load resistance. We utilize silicon steel CT core (23JGSD085, JFE Steel Corp, Tokyo, Japan), a widely used material known for its high magnetic permeability and low energy loss. The load current tester is customized, and the current input range lies between 0 A and 70 A. The DSOX3024T oscilloscope and the N2821A current probe with a bandwidth of 200 MHz, made by KEYSIGHT Technologies Inc. (Santa Rosa, CA, USA), are used for the experimental analysis. A cement resistor with an input resistance range of 20 Ω to 1000 Ω made by Baro Electronics Co. (Yongin, Republic of Korea) is used as the load resistance. The experiments were conducted at room temperature (approximately 25 °C).

To collect the experiment data, the load current tester applied current to the powerline, generating a magnetic field that magnetized the CT core and induced voltage and current. The coil connected to the core was coupled with a resistance and the induced voltage and current were measured under varying resistance conditions using a current probe and oscilloscope. The collected data were then used to calculate the power generated by the CT core.

### 2.2. Numerical Simulation

Ansys Maxwell 3D (Ansys, Inc., Canonsburg, PA, USA) is employed to simulate the electromagnetic behavior of a CT core. We obtained the distributions of the magnetic flux, induced voltages, and induced currents to evaluate the performance of the CT core. The calculation is based on the four Maxwell equations (Ampere’s law, Gauss’s law of magnetism, Faraday’s law, and Gauss’s law of electricity), given as follows:

(1) $$\nabla \times H=J+\frac{\partial D}{\partial t}$$

(2) ∇×B=0

(3) $$\nabla \times E=-\frac{\partial B}{\partial t}$$

(4) ∇×D=ρ

As shown in Figure S1, for the numerical simulation, the CT core comprises the following sizes: the outer radii (Ro) are 45 mm, 50 mm, 55 mm, and 62.5 mm, while the inner radii (Ri) are 35 mm, 37.5 mm, and 40 mm. The heights (h) are 55 mm, 60 mm, and 65 mm, respectively. The coil is set to 100 turns. The minor radius and major radius of the coil are 0.7 and 40 mm, respectively. The air gap range of the split core is applied to 2 μm, 4 μm, 6 μm, 8 μm, and 10 μm.

The powerline is positioned at the center of the CT core, with a radius of 1 mm and a height of 100 mm. The coil and powerline are composed of copper, and the core material is composed of silicon steel.

### 2.3. Polishing of the Split Core

To polish the cross-section of the split core, firstly the ring core was processed to split core using water-jet cutting. Subsequently, a surface grinding machine is used to polish the rough surface formed by the water-jet cutting and to smooth out any visually uneven sections through two times of the wet polishing process. Finally, buffing is performed to eliminate minor irregularities in the cross-section and to achieve a polished split core through two times of fine-polishing process. During the first fine-polishing process, uneven surfaces are polished, and in the second fine-polishing process, the grinding stone is lowered horizontally by 0.05 mm before buffing.

### 2.4. Surface Roughness Measurement

Air gaps caused by surface roughness can break the magnetic circuit, leading to a decrease in core performance. In order to investigate the influence of the air gap on the core performance of the air gap surface, the surface roughness of the core cross-section was measured using a profilometer. The profilometer is NPFLEX 3D optical profiler (Bruker Corp., Billerica, MA, USA) with the resolution of less than 0.1 nm. Two split cores (i.e., eight cross-section surfaces) were used in the measurements, with three points measured on each cross-section.

In order to correct for the occurring gradient during measurement of specimens, the surface equation of the raw data are obtained using linear regression analysis, it can be expressed as Z = b_0_ + b_1_ ×X + b_2_ × Y, where b_0_, b_1_ and b_2_ are the coefficients and are obtained with MATLAB R2023b.

To obtain the corrected surface roughness, the subtraction between original height and mean height is calculated and the values are adjusted again to align with the absolute coordinates of the surface roughness distribution.

## 3. Results and Discussion

**Figure 1:**
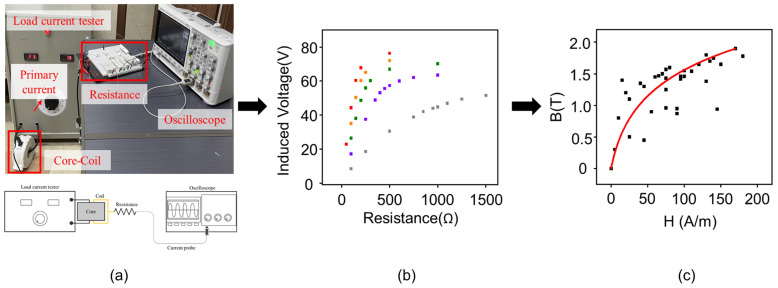
B-H curve estimation workflow. (**a**) Experimental setup to measure voltage-resistance data (**b**) voltage data corresponding to different resistances for B-H curve estimation at various currents. (**c**) analytically obtained B-H curve with trendline.

**Figure 2:**
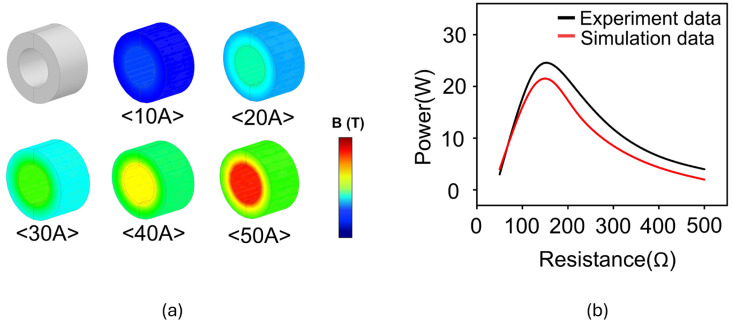
Numerical simulation based on obtained B-H curve to estimate the core performance, e.g., (**a**) magnetic flux density, corresponding to the current. (**b**) Comparison of power performance between experiment and simulation data.

**Figure 3:**
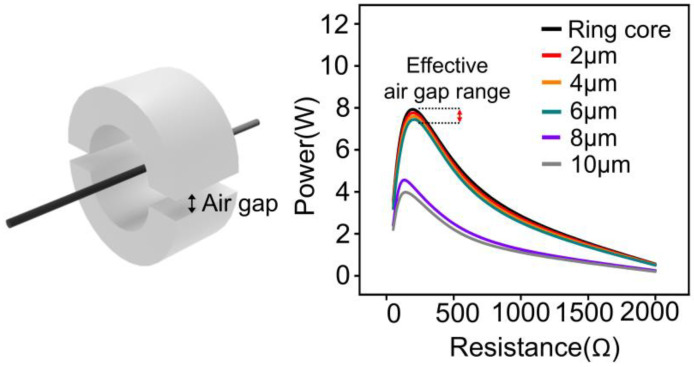
Design of an appropriate air gap range of a split core for the required performance.

Figure 1, Figure 2 and Figure 3 depict the overall procedure to obtain the B-H curve of the CT core and to predict its various performances based on the obtained B-H curve. Firstly, the B-H curve was obtained using the trend line from the experimental results (Figure 1). As shown in Figure 1a, when a primary current is applied to the core and the coil of the CT core by a load current tester, the core becomes magnetized due to the magnetic field formed around the primary current. This magnetization induces a secondary current in the coil. Subsequently, a voltage is generated while the secondary current passes through the load resistance. This voltage can be measured using an oscilloscope to obtain the voltage data for each resistance where each color represents a different applied current condition (Figure 1b). Lastly, the B-H curve was obtained in the form of a trendline based on the voltage data for various current and resistance conditions (Figure 1c). In addition, an electromagnetic field analysis was performed for various current and resistance conditions by using the derived B-H curve. The performance of the core, such as the distribution of magnetic flux density and power, can be analyzed based on this analysis (Figure 2). On the other hand, the split core must exhibit a lower performance when compared to the ring cores due to the inevitable presence of an air gap if they are under the same conditions.

An appropriate air gap must be considered in the design of a split core to overcome this performance degradation. Thus, the performance of the split core was analyzed for various air gap ranges in comparison with that of the ring cores, and the air gap range was validated to minimize the difference in the performance between the split and ring cores (Figure 3).

**Figure 4:**
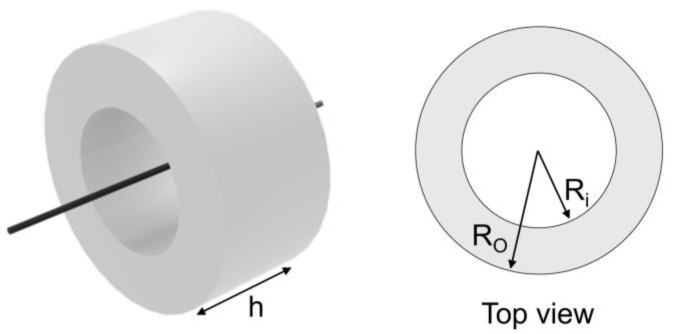
Schematic diagram of the ring core. Ring core was designed with a R_i_, R_o_, and h.

**Figure 5:**
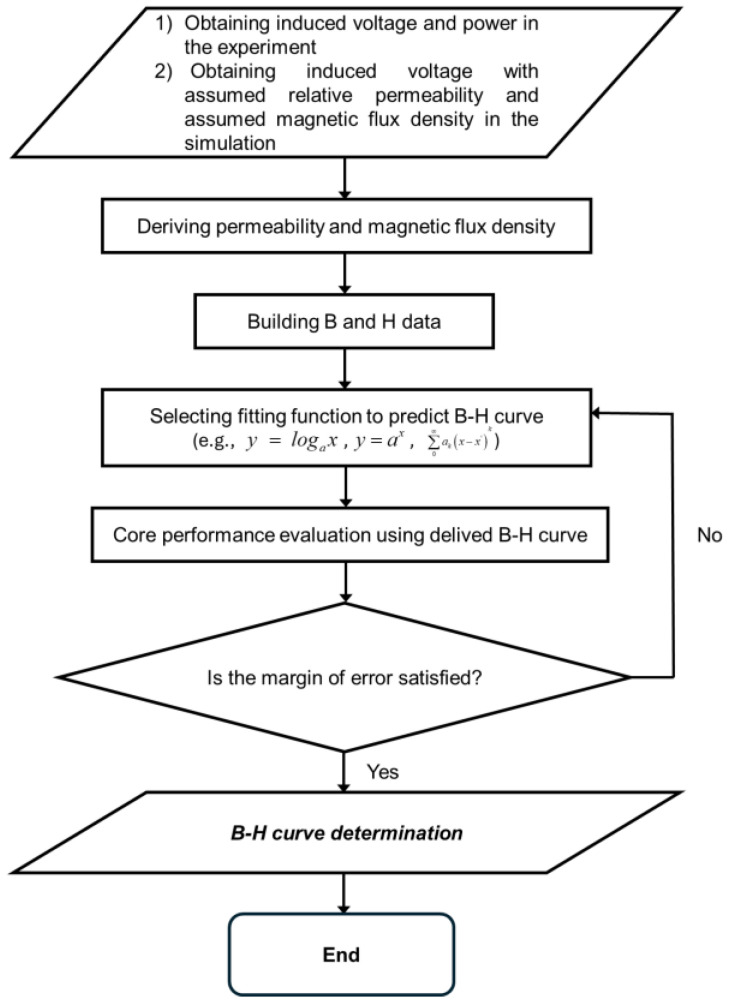
Process for estimating B-H curve.

**Figure 6:**
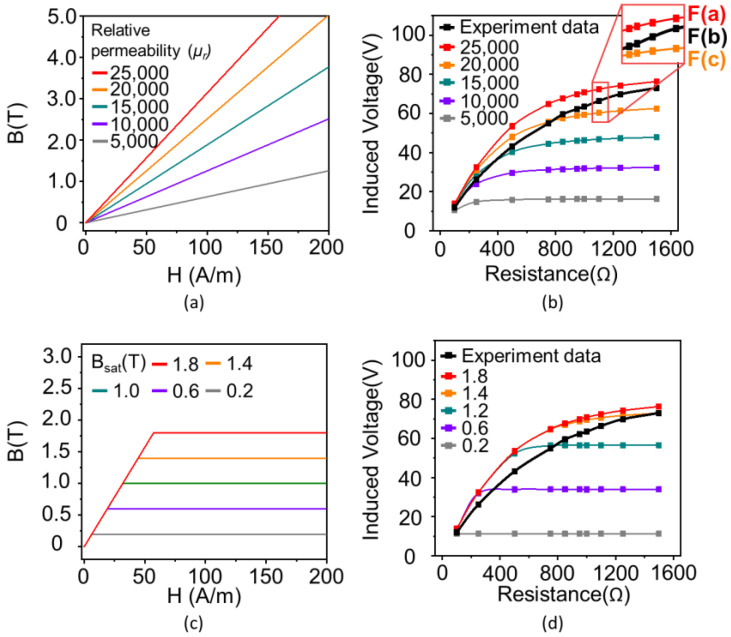
Procedure for obtaining permeability and magnetic flux density of considered core using interpolation. (**a**) Various assumed relative permeabilities required to simulate electromagnetic behavior of core. (**b**) Obtained induced voltage with assumed permeabilities and interpolation to determine permeability under each resistance. (**c**) Various assumed magnetic flux densities, B_a_, required to simulate core electromagnetic behavior. (**d**) Obtained induced voltage with assumed B_a_ and interpolation to determine B under each resistance.

**Figure 7:**
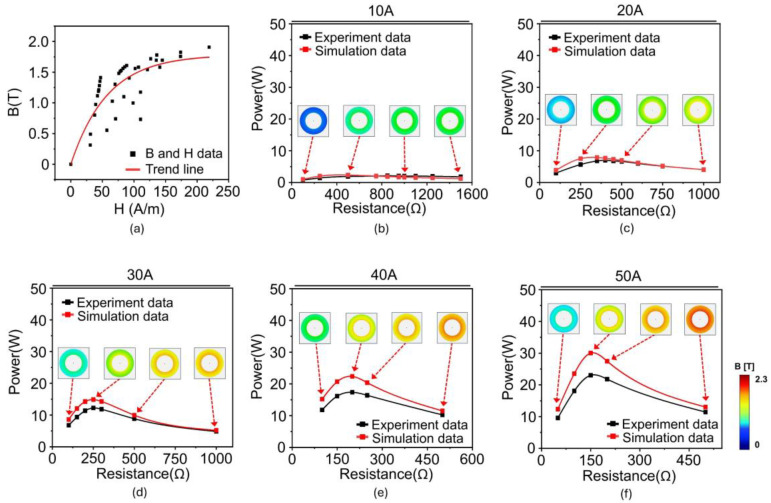
(**a**) Analytically estimated B-H curve of silicon steel core with trendline based on B and H data of all the currents. (**b**–**f**) Comparison of power vs. resistance performance of core between simulation and experiment at 10, 20, 30, 40, and 50 A, respectively. Insets presented the distribution of magnetic flux density (B) inside the core over a current range of 10 A to 50 A. Color corresponds to the magnetic flux density within core.

**Figure 8:**
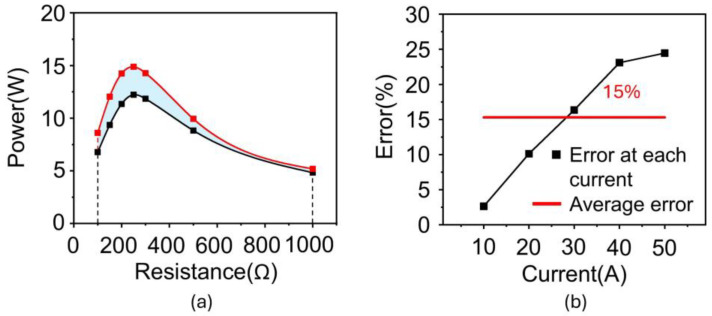
Validation of obtained silicon steel B-H curve. (**a**) Difference of area under a curve of power vs. resistance. (**b**) Errors between simulation and experiment.

**Figure 9:**
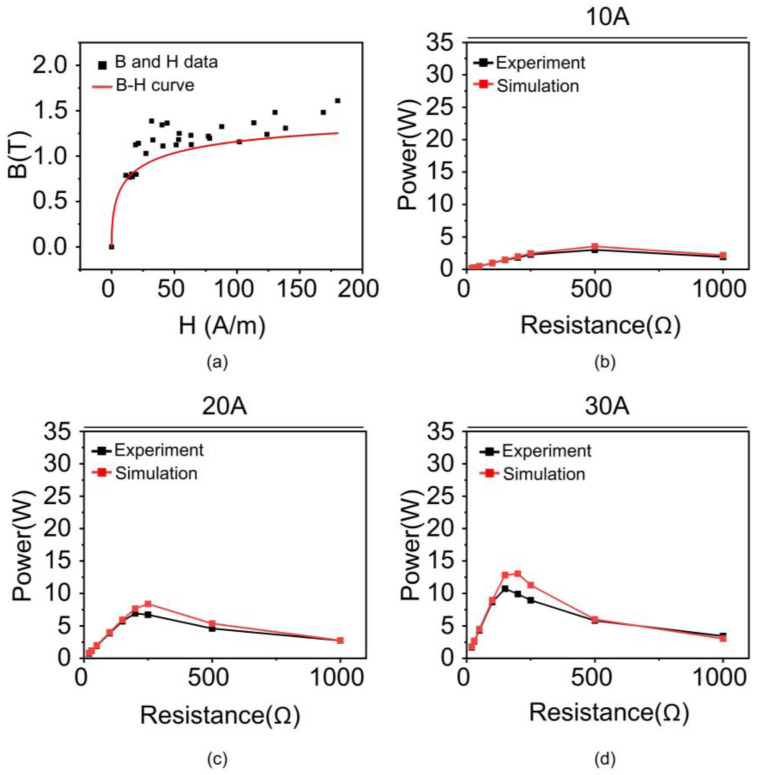
(**a**) Analytically estimated B-H curve of nanocrystal core with trendline based on B and H data of all the currents (**b**–**d**) Comparison of power vs. resistance performance of core between simulation and experiment at 10, 20, and 30 A, respectively.

**Figure 10:**
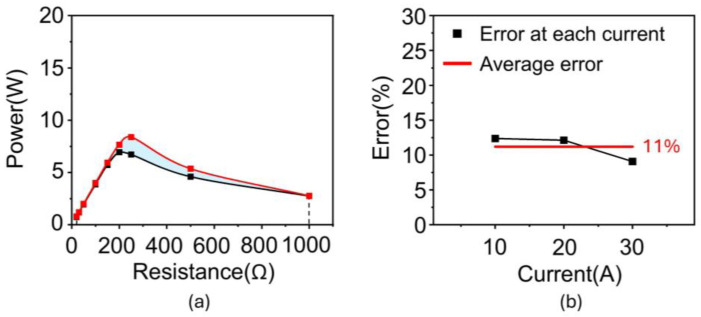
Validation of obtained nanocrystal B-H curve. (**a**) Difference of area under a curve of power vs. resistance. (**b**) Errors between simulation and experiment.

Figure 4, Figure 5, Figure 6, Figure 7, Figure 8, Figure 9 and Figure 10 show the estimated B-H curve for the ring core (i.e., the CT core) and its validation to assess the core performance. An electromagnetic simulation (ANSYS Maxwell 3D 2021 R2) was conducted, and the B-H curve was estimated for various input currents using the analytically derived B and µ values. To verify the performance of the obtained B-H curve, we evaluate the error between the power obtained through simulation under the same current and resistance conditions as the experiment, and the power measured in the actual experiment. The ring core in the simulation was modeled as circular, which is made of silicon steel, with a 62.5 mm R_o_, a of 37.5 mm R_i_, and a 60 mm h (Figure 4). The applied input currents ranged from 10 A to 50 A, and a winding of 100 turns was applied at a frequency of 60 Hz. A flowchart for estimating B-H curve is shown in Figure 5.

Firstly, to estimate the B-H curve, the induced voltage and power data exhibited by the CT core under various current and resistance conditions are experimentally measured. Subsequently, in the simulation, assumed relative permeability and magnetic flux density are applied to derive the induced voltage. Then, by comparing the induced voltage obtained in the experiment with that obtained in the simulation, the relative permeability and magnetic flux density corresponding to each current and resistance condition are determined through interpolation. The magnetic fields are calculated using Equation (6). This iterative process generates a dataset of magnetic flux density and magnetic field. Finally, by selecting an appropriate fitting function that reflects the trend of the generated magnetic flux density and magnetic field data, the B-H curve is constructed. The accuracy of the B-H curve is validated by comparing the power obtained in the simulation under the same current and resistance conditions with the experimentally measured power. If the evaluated error exceeds the user-defined acceptable range, the fitting function is reselected, and the process is repeated. In this study, we set the average error to within 15%, and based on this criterion, we ultimately obtained a B-H curve that showed errors within the acceptable range under various current and resistance conditions. The detailed explanations are provided in Figure 6.

Figure 6a,b depict the procedure used to determine the relative permeability, µ_r_, for the considered core when the input current is 10 A. The values of the relative permeability, µ_r_a_, which were assumed as 5000, 10,000, 15,000, 20,000, and 25,000, were applied in the simulation and induced voltage data were obtained with µ_r_a_. The obtained voltage data were compared with the experimental data. µ_r_ of the considered core under each resistance condition can be derived using Equation (5), through interpolation based on two specified points, as shown in the inset of Figure 6b.

(5) $$F\left(b\right)=F\left(c\right)+\left[\frac{F\left(a\right)-F\left(c\right)}{a-c}\right]\left(b-c\right)$$

Similarly, the values of magnetic flux density, B_a_, were assumed to be set to 0.2, 0.6, 1.0, 1.4, and 1.8 T to obtain the magnetic flux density, B, as shown in Figure 6c,d. The induced voltage was obtained using B_a_ and compared with the experimental data, where µ_r_ was set to 25,000. B of the core can be derived for each resistance by using Equation (5). For example, µ was calculated as 0.009165 H/m, and B was calculated as 0.283995 T at 100 Ω. Using the obtained µ and B, the magnetic field H was calculated using Equation (6), which describes the correlation between B and H. µ_0_ denotes the permeability of free space and has a correlation, µ = µ_r_µ_0_, with the permeability, µ.

(6) $${B=\mu }_{r}\mu_{0}H$$

Table 1 lists the results of the derived µ, B, and H for 10 A. The same procedure was performed to obtain the B and H data from 20 A to 50 A (Tables S1–S4). All the B and H data for all the input currents (i.e., 10, 20, 30, 40, and 50 A) were plotted on a single graph (Figure 7a). Lastly, a unified B-H curve was obtained from the trend line of the data. Figure 7b–f depicts the power of the silicon steel core from the simulation using a unified B-H curve with an input current ranging from 10 A to 50 A. All the results exhibit similar values to the experimental data. Insets presented the distribution of magnetic flux density (B) inside the core over a current range of 10 A to 50 A. As shown in the insets, when the current increases, the magnetic flux density in the core increases because the magnetic field around the power line becomes stronger. Additionally, as resistance increases, the voltage increases, which means the rate of change of magnetic flux increases. These changes lead to a stronger distribution of magnetic flux density inside the core. As the current increases, the magnetic field around the power line becomes stronger, and correspondingly, the distribution of magnetic flux density also tends to increase. As the resistance increases, the voltage increases according to Ohm’s law, which in turn increases the rate of change of magnetic flux according to Faraday’s law. This results in a stronger formation of magnetic flux density in the core, impacting its performance. The effectiveness of the obtained B-H curve was validated by calculating the error between the simulation and experiment, which was obtained by the difference in the areas under the curve (Figure 8a). Consequently, the error exhibits only 2.6% under 10 A and was estimated to be approximately 15% in all cases, indicating that the estimated B-H curve effectively reflects the magnetization characteristics of the silicon steel core (Figure 8b).

Additionally, we evaluated the accuracy of the estimation model using widely used standard metrics such as Mean Absolute Error (MAE), Mean Square Error (MSE), and Root Mean Square Error (RMSE). As observed in all metrics, the error decreases as the current decreases, and the lowest error is observed at 10 A (Table 2). To verify the applicability of the proposed B-H curve estimation method to different materials, we additionally investigated the performance of the B-H curve derived using the same method when the core material was considered as nanocrystal. The same core geometry as that of silicon steel was considered. The obtained B-H curve and comparison of core performance between simulation and experiment are shown in Figure 9. The results show that the power performance predicted by the simulation using the derived B-H curve was estimated to be approximately 11% in all cases, indicating that the estimated B-H curve effectively reflects the magnetization characteristics of the nanocrystal core, as compared to the power performance obtained from the experiments (Figure 10). Additionally, we evaluated the accuracy of nanocrystal B-H curve using widely used standard metrics, which showed that the power results from the simulation and the experiments were very similar (Table 3).

**Figure 11:**
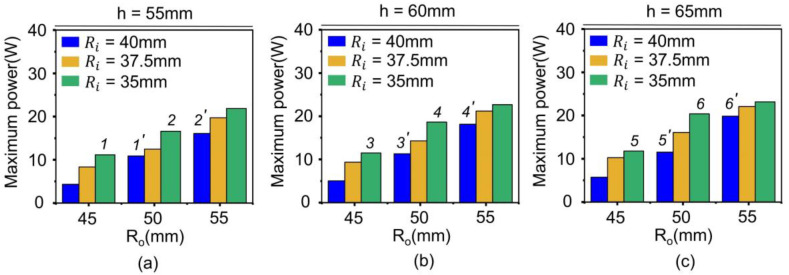
Comparison of maximum power vs. R_o_ of three different ring cores with R_i_ of 35 mm, 37.5 mm, and 40 mm in simulation for h of (**a**) 60 mm, (**b**) 65 mm, and (**c**) 70 mm, respectively. Cases with indices (e.g., 1 and 1’) mean a reversal in performance despite larger volume.

**Figure 12:**
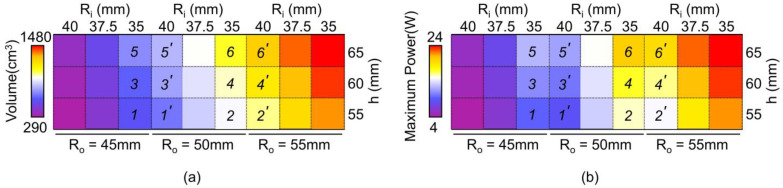
(**a**) Volume map profile for various values of R_o_, R_i_, and h. (**b**) Maximum power map profile for various values of R_o_, R_i_, and h.

**Figure 13:**
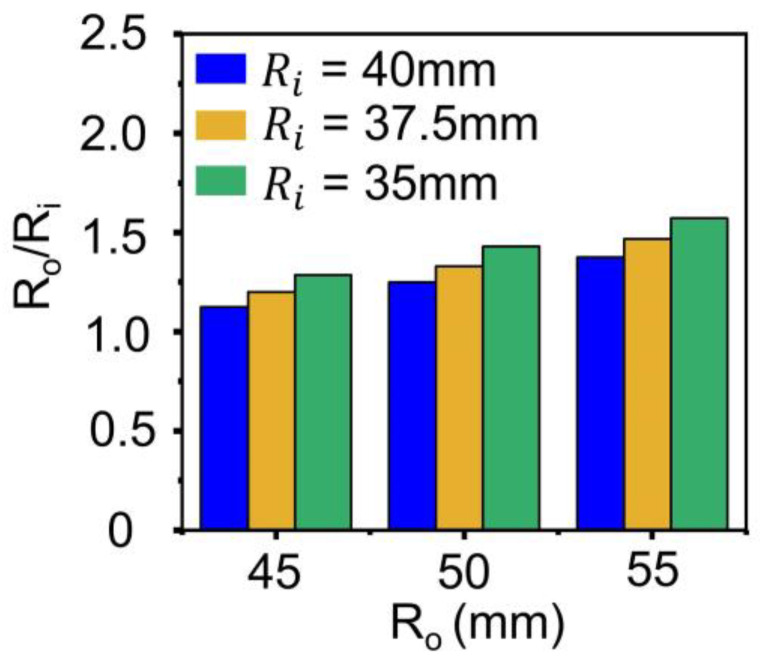
Comparison of R_o_/R_i_ vs. R_o_ of three ring cores with R_i_ of 35 mm, 37.5 mm, 40 mm.

Figure 11, Figure 12 and Figure 13 depict the effect of core geometry on the performance. To analyze this effect, the core was modeled by using shape parameters, such as R_o_, R_i_, and h. The simulation was performed using different values of R_o_ (45, 50, and 55 mm), R_i_ (35, 37.5, and 40 mm), and h (55, 60, and 65 mm), and the B-H curve obtained from Figure 8a was applied. Figure 11 shows the maximum power based on the R_o_, R_i_ and h of the core. When the h of the core was 55 mm, the maximum power increased with the increase in R_o_ and R_i_. Figures S2–S4 depict the power results for all the core geometries. These results demonstrate a similar tendency when h was 60 mm and 65 mm. However, interestingly, the labeled cases (i.e., 1-1’~6-6’) exhibited reverse results at maximum power.

To investigate the reason for the reversal results, core volume was calculated and compared to the maximum power (Figure 12). The core volume gradually increased with the increase in R_o_, R_i_, and h, as shown in Figure 12a. In other words, those labeled cores show that a core with a small volume has a higher power. For example, 1 (11.151 W) has higher power than 1’ (10.904 W), even though 1 (552.920 cm^3^) has a smaller volume than 1’ (662.035 cm^3^), as shown in Figure 12. This implies that the power of the core does not simply depend on the core volume. These results can be explained by the induced voltage, which depends on various other variables rather than the volume alone, as shown in Equation (7).

(7) $$V=N\times \mu \times h\times f\times I\times ln(\frac{Ro}{Ri})$$

Figure 13 depicts the ratio of the outer radius to the inner radius, R_o_/R_i_, demonstrating a similar increase in power. Among the variables in Equation (7), the h of core, and the R_o_/R_i_ correspond to the geometry of the core. Where N is the number of turns in the coil, µ is relative permeability, h is the height of the ring core, f is the frequency of the power line, and I is the power line current. The increase in power within each graph corresponding to R_o_/R_i_ was sharper than that corresponding to h as shown in Figure 11. This indicates that R_o_/R_i_ is more dominant in core performance among the geometry variations of the core.

**Figure 14:**
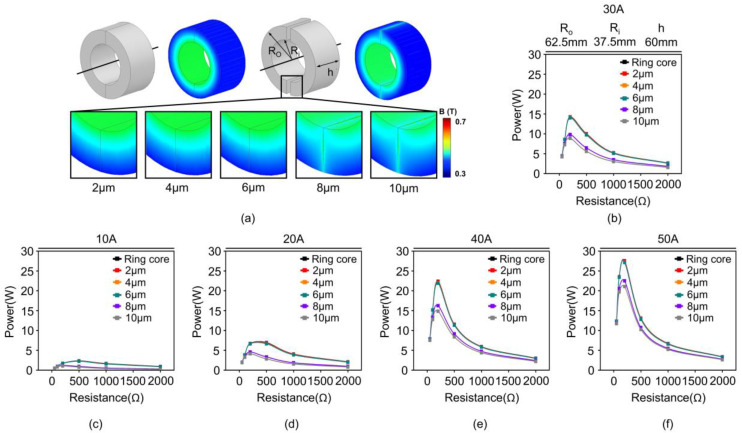
(**a**) Distribution of magnetic flux density in ring core and split cores with air gaps of 2 μm, 4 μm, 6 μm, 8 μm, and 10 μm. The color corresponds to the magnetic flux density in core (**b**) Comparison of power vs. resistance performance between ring core and split core with air gaps of 2 μm, 4 μm, 6 μm, 8 μm, and 10 μm at 30 A. (**c**–**f**) Comparison of power vs. resistance performance between ring core and split core with air gaps of 2, 4, 6, 8, and 10 μm at 10, 20, 40, and 50 A.

Subsequently, an appropriate range of air gaps to design the split core was analyzed using the obtained B-H curve, which could provide similar power to the ring core based on the difference in performance between the ring and split cores. Figure 14a shows the magnetic flux density distribution of the ring core and split core with a R_o_ of 62.5 mm, R_i_ of 37.5 mm, and h of 60 mm when the input current was 30 A. The air gap of the split core was applied from 2 μm, 4 μm, 6 μm, 8 μm, and 10 μm, respectively. The magnetic flux density of the split core with an air gap under 6 μm was distributed uniformly along the circular core shape, which is similar to that of the ring core. However, the magnetic circuit break corresponding to the air gap increased when the air gap of the split core exceeded 6 μm. The magnetic flux density increased on the air gap surface and exhibited a non-uniform distribution over this range of air gaps. Since the relative permeability of air is substantially lower compared to that of the magnetic core material, the air gap introduced a significant increase in magnetic reluctance within the air gap region as the air gap size grew [52,53]. This non-uniform distribution was caused by this increase in magnetic reluctance, which further disrupted the magnetic circuit, reducing the effective permeability and limiting the flow of magnetic flux through the core. Consequently, the performance of the split core degraded, and this degradation became more severe as the air gap size continued to increase.

Figure 14b–f depicts the power according to the input currents ranging from 10 A to 50 A of a split core with air gaps ranging from 2 to 10 μm and that of the ring core. The graph shows that both the split core with an air gap below 6 μm and the ring core achieved a maximum power output of approximately 14 W. However, the split cores with air gaps of 8 μm and 10 μm exhibited lower values of 9.8 W and 8.9 W, respectively. The split cores with air gaps of less than 6 μm presented a similar performance to the ring core, whereas a significant decrease in the performance was observed in split cores with air gaps of 8 μm or more. This decrease can be attributed to the clear interruption of the magnetic flux within the core, as shown by the magnetic flux density distribution in Figure 14a, when an air gap of 8 μm or greater is present, resulting in significant performance degradation.

Figure 14a,b demonstrate that the air gap must be minimized (i.e., to less than 6 μm) to ensure that the performance is equivalent to that of the ring core. The cross-section of the split core was polished to achieve this effect.

**Figure 15:**
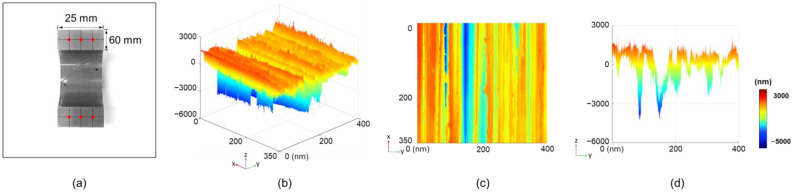
(**a**) Cross-section image of the split core after the polishing process and (**b**) 3D surface roughness distribution of the corresponding cross-section. (**c**) Top view of the surface roughness distribution. (**d**) Side view of the surface roughness distribution with a color bar indicating the roughness range from −5000 nm to 3000 nm.

**Figure 16:**
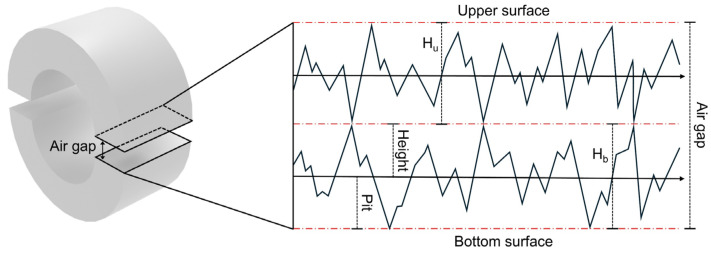
Schematic image of air gap range due to surface roughness in split core.

The surface roughness of the split core cross-section was measured to analyze the surface properties of the polishing, as shown in Figure 15a. Figure 15b–d shows the distribution of surface roughness on the cross-section of the core used in the experiment. The size of the air gap was defined by the height and pit length. The maximum height of each surface was determined as the sum of the height and pit length. The air gap was defined as twice the maximum height because each surface was in contact with the split core (Figure 16). For example, the air gap of 2 μm indicates that the upper and bottom components of the split core have a maximum height of 1 μm, respectively. The surface roughness distribution for each point on the core is measured and the average distribution of the total cross-section surface (i.e., air gap) is listed in Table S5. Consequently, the ratio of air gaps of 2 μm or more in the measured surface roughness data was approximately 12.3%. Similarly, the ratio of air gaps of 4 μm or more was 2.57%, 6 μm or more was 0.846%, 8 μm or more was 0.338%, and 10 μm or more was 0.163%.

**Figure 17:**
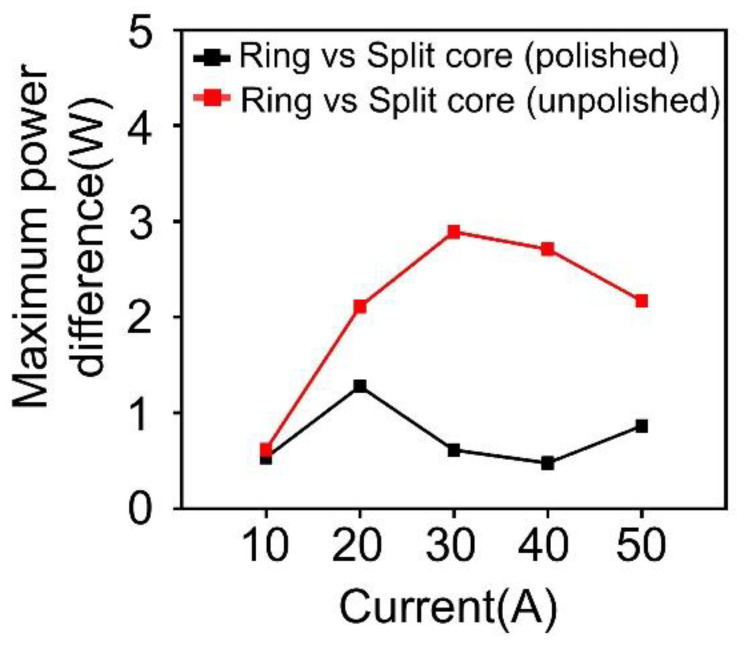
Comparison of maximum power difference between the ring core and the split cores (i.e., polished and unpolished) according to the current variation in the experiment.

Figures S5 and S6 present the distribution of surface roughness before polishing. As shown in Table S6, the split core before polishing has a rougher surface to generate a larger air gap over 6.0 μm and it has a significant difference of power with ring core. These results indicate that the air gap of the split core can be formed within a range of 6 μm or less after the polishing process. Figure 17 presents a comparison of the experimentally measured power between the ring core and the split cores (i.e., polished and unpolished). As predicted by the simulation, it can be observed that the split core with the air gap formed in the range of 6 μm or less through polishing, exhibits a performance more similar to the ring core than the unpolished split core.

**Figure 18:**
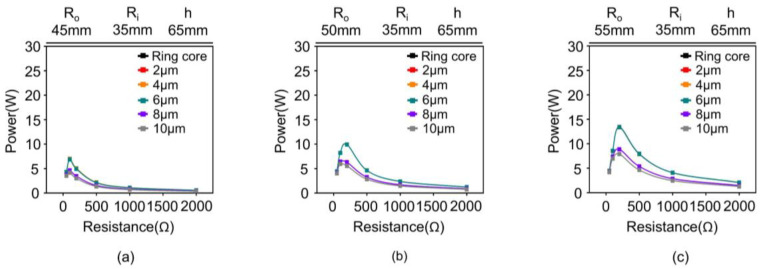
Comparison of power vs. resistance performance between ring core and split core of 2 μm, 4 μm, 6 μm, 8 μm, and 10 μm. The R_o_ are: (**a**) 45 mm, (**b**) 50 mm, and (**c**) 55 mm. The R_i_ and h are 35 mm and 65 mm, respectively.

Figure 18 depicts the effect of the air gap on various core geometries. The simulation was performed using three different R_o_ (i.e., 45, 50, and 55 mm), an R_i_ of 35 mm, and a h of 65 mm. In all the cases, the performance of a split core with an air gap below 6 μm presents a performance similar to that of the ring core. However, as the R_o_ of the core increased, the performance degradation tended to increase with the increase in the air gap.

The increase in the area of the air gap is attributed to the increase in the core size. These results indicate that an air gap of approximately 6 μm is the effective range for achieving performance comparable to that of a ring core.

In this study, we proposed an intuitive method to estimate the B-H curve of the CT core using resistance-voltage data obtained under various current and resistance conditions. The method achieved an average error of 15%, with the most accurate cases showing an error as low as 2.6%. Additionally, this approach eliminates the need for expensive equipment such as VSM or Epstein, which are traditionally required for B-H curve derivation. Our findings highlight the importance of air gap optimization in split cores, demonstrating that maintaining an air gap below 6 μm ensures performance comparable to that of ring cores.

While this method simplifies the B-H curve estimation process, slight errors may occur due to data dispersion during interpolation. To address this, non-linear interpolation techniques could be used to enhance the accuracy of B and H data collection. Furthermore, incorporating core loss models—such as those accounting for hysteresis and eddy current effects—into finite element (FE) simulations could further reduce errors and improve the alignment between experimental and numerical results, even though core losses are generally negligible under low-frequency conditions in transformers. Additionally, selecting fitting functions that appropriately reflect the experimental data is crucial for improving B-H curve accuracy. Enhancing the accuracy of the B-H curve through carefully chosen fitting functions is directly tied to the design of high-performance CT cores.

Building on the identified limitations of this study, future research should aim to address these challenges to enhance the accuracy and applicability of B-H curve estimation methods and further improve CT core performance. By pursuing these directions, future studies can refine the methods proposed here, contributing to the development of more accurate and efficient energy harvesting devices based on CT cores.

## 4. Conclusions

In this study, we proposed an intuitive method to estimate the B-H curve of the CT core and demonstrated its validity by comparing the CT core performance between the numerical and experimental results. Additionally, the effective range of the air gap was analyzed to design the split core, which can minimize the performance degradation due to the air gap by using the obtained B-H curve.

Electromagnetic field simulations were conducted using the analytically derived B-H curves based on the experimentally measured resistance-voltage data. The simulation results demonstrated that the predicted performance of the CT core had an error of only 2.6%, compared to the experimentally observed performance under the 10 A condition, which is the most accurate case. This highlights the significance of the proposed method for estimating the B-H curves, which is simpler and more intuitive than that of the existing methods.

The effect of the CT core’s shape on its performance was analyzed along with the obtained B-H curve. The simulation results indicated that the core’s performance is not dependent on its volume alone, and is also influenced by the ratio of its inner and outer radii.

On the other hand, the split cores experience a decrease in performance when compared to the ring cores due to the inevitable presence of an air gap. Minimizing this gap is crucial, and simulations predicted that the split cores could achieve a performance similar to that of the ring cores when the air gap is within a range of 6 μm or less. The split cores that demonstrated similar performance to the ring cores were experimentally validated by conducting surface polishing on their cross-section, ensuring surface roughness within the range of 6 μm.

In summary, we have proposed a highly effective and straightforward method to derive the B-H curve, which is crucial in predicting and evaluating the performance of CT cores. To the best of our knowledge, this method has not been reported previously. Although the proposed method exhibits relatively large errors in some cases, they can be improved by using more resistance-voltage data and advanced interpolation techniques when deriving the final B-H curve.

The proposed analysis method, along with the prediction and estimation of CT core performance, presents considerable potential for designing high-performance CT cores. These findings could potentially lead to the development of versatile design systems for energy harvesting devices.

## Figures and Tables

**Table 1:** Simulation data for 10A.

Resistance (Ω)	B (T)	µ (H/m)	H (A/m)
100	0.312	0.01	30.898
250	0.49	0.016	31.165
500	0.802	0.021	37.856
750	0.974	0.025	39.399
850	1.114	0.026	42.197
950	1.187	0.027	43.799
1000	1.212	0.027	44.305
1100	1.28	0.028	45.251
1250	1.35	0.029	46.12
1500	1.411	0.03	47.181

**Table 2:** Silicon steel core performance using various metrics.

Current (A)	MAE	MSE	RMSE
10	0.38	0.17	0.41
20	0.62	0.7	0.89
30	2	4.76	2.04
40	3.64	14.94	3.87
50	4.48	24.1	4.91

**Table 3:** Nanocrystal core performance using various metrics.

Current (A)	MAE	MSE	RMSE
10	0.14	0.05	0.22
20	0.41	0.44	0.66
30	0.98	2.22	1.49

## Data Availability

The original contributions presented in the study are included in the article; further inquiries can be directed to the corresponding author (J.S.).

## Supplementary Materials

The following supporting information can be downloaded at: https://www.mdpi.com/article/10.3390/ma18030644/s1, Table S1: Simulation data for 20A; Table S2: Simulation data for 30A; Table S3: Simulation data for 40A; Table S4: Simulation data for 50A; Table S5: Air gap distribution of total cross-section surfaces after polishing; Table S6: Air gap distribution of total cross-section surfaces after polishing; Figure S1: Schematic image of the core geometrical parameters for numerical simulation. (a) Geometric parameters of the split core, including the outer radius (Ro), inner radius (Ri), and height (h). Major and minor radius of the coil are shown, with Ro = 45, 50, 55, 62.5 mm, Ri = 35, 37.5, 40 mm, h = 55, 60, 65 mm, Major radius = 40 mm, and Minor radius = 0.7 mm. (b) Visualization of split core air gaps with numerical simulations for air gap sizes of 2 µm, 4 µm, 6 µm, 8 µm, and 10 µm, illustrating the progressive enlargement of the air gap; Figure. S2: Comparison of power vs. resistance performance of various core geometry with R_i_ of 35 mm, 37.5 mm, and 40 mm, h of 55 mm, 60 mm, and 65 mm in simulation for the R_o_ of (a) to (i) 45 mm, respectively; Figure S3: Comparison of power vs. resistance performance of various core geometry with R_i_ of 35 mm, 37.5 mm, and 40 mm, h of 55 mm, 60 mm, and 65 mm in simulation for the R_o_ of (a) to (i) 50 mm, respectively; Figure S4: Comparison of power vs. resistance performance of various core geometry with R_i_ of 35 mm, 37.5 mm, and 40 mm, h of 55 mm, 60 mm, and 65 mm in simulation for the R_o_ of (a) to (i) 55 mm, respectively; Figure S5: Surface roughness distribution before polishing process. (a) 3D surface roughness distribution of the corresponding cross-section. (b) Top view of the surface roughness distribution. (c) Side view of the surface roughness distribution with color bar indicating the roughness range from −25000 nm to 20000 nm.
