# Supplementary material for: B-H Curve Estimation and Air Gap Optimization for High-Performance Split Core
Source: Materials (Basel). 2025 Jan 31;18(3):644. doi: 10.3390/ma18030644 (PMC11820830; doi:10.3390/ma18030644)
Supplement: Supplementary file 1 [file materials-18-00644-s001.zip › materials-3423042-supplementary.pdf]

Supplementary Material for the manuscript

B-H Curve Estimation and Air Gap Optimization for High-Performance Split Core

Minjoong Kim [1,†], Myungseo Lee [1,†], Sijeong Lee [1], Jaeyun Lee [2,*] and Jihwan Song [1,*]

[1] Department of Mechanical Engineering, Hanbat National University, 125 Dongseodae-ro, Yuseong-gu, Daejeon 34158, Republic of Korea; kimminjoong.mmm@gmail.com (M.K.); myungseo.mmm@gmail.com (M.L.)
[2] Corechips, 33 Omokcheon-ro Gwonseon-gu, Suwon 16642, Republic of Korea
* Correspondence: jylee@corechips.co.kr (J.L.); jsong@hanbat.ac.kr (J.S.)
† These authors contributed equally to this work.

Table S1. Simulation data for 20A.

Resistance(Ω)	B(T)	μ(H/m)	H(A/m)
100	0.554	0.01	57.165
250	1.028	0.016	65.709
350	1.303	0.019	70.046
400	1.481	0.019	76.022
450	1.512	0.019	78.208
500	1.528	0.019	79.949
600	1.56	0.019	82.627
750	1.586	0.019	85.47
1000	1.609	0.018	88.434

Table S2. Simulation data for 30A.

Resistance(Ω)	B(T)	μ(H/m)	H(A/m)
100	0.741	0.01	70.911
150	1.1	0.013	83.955
200	1.403	0.015	92.551
250	1.559	0.015	101.802
300	1.58	0.015	107.158
500	1.718	0.014	126.548
1000	1.779	0.013	136.124

Table S3. Simulation data for 40A.

Resistance(Ω)	B(T)	μ(H/m)	H(A/m)
100	0.998	0.01	98.579
150	1.543	0.013	121.331
200	1.694	0.013	135.328
250	1.694	0.012	144.911
500	1.824	0.01	173.78

Table S4. Simulation data for 50A.

Resistance(Ω)	B(T)	μ(H/m)	H(A/m)
50	0.732	0.007	110.471
100	1.175	0.011	110.494
150	1.58	0.011	140.957
200	1.757	0.01	173.776
500	1.907	0.009	218.973

Table S5. Air gap distribution of total cross-section surfaces after polishing.

Air gap (μm)	Percentage relative to the total data (%)
≤ 2.0	87.7
≥ 2.0	12.3
≥ 4.0	2.57
≥ 6.0	0.846
≥ 8.0	0.338
≥ 10.0	0.163

Table S6. Air gap distribution of total cross-section surfaces after polishing.

Air gap (μm)	Percentage relative to the total data (%)
≤ 2.0	10.699
≥ 2.0	89.301
≥ 4.0	78.799
≥ 6.0	68.709
≥ 8.0	59.125
≥ 10.0	50.160

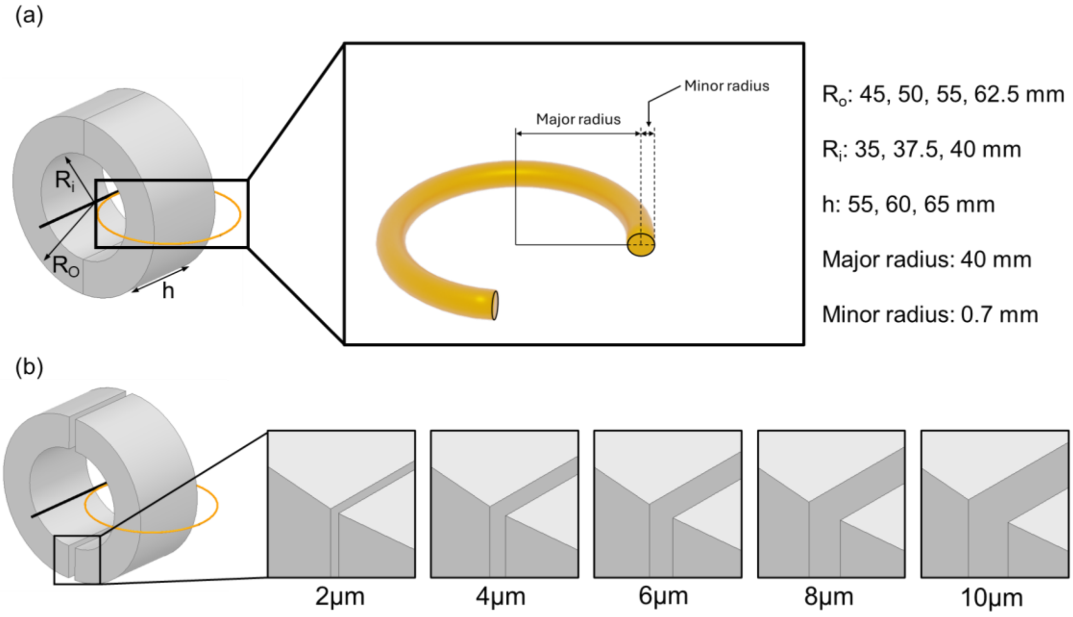

Figure S1. Schematic image of the core geometrical parameters for numerical simulation. (a) Geometric parameters of the split core, including the outer radius (Ro), inner radius (Ri), and height (h). Major and minor radius of the coil are shown, with Ro = 45, 50, 55, 62.5 mm, Ri = 35, 37.5, 40 mm, h = 55, 60, 65 mm, Major radius = 40 mm, and Minor radius = 0.7 mm. (b) Visualization of split core air gaps with numerical simulations for air gap sizes of 2 μm, 4 μm, 6 μm, 8 μm, and 10 μm, illustrating the progressive enlargement of the air gap.

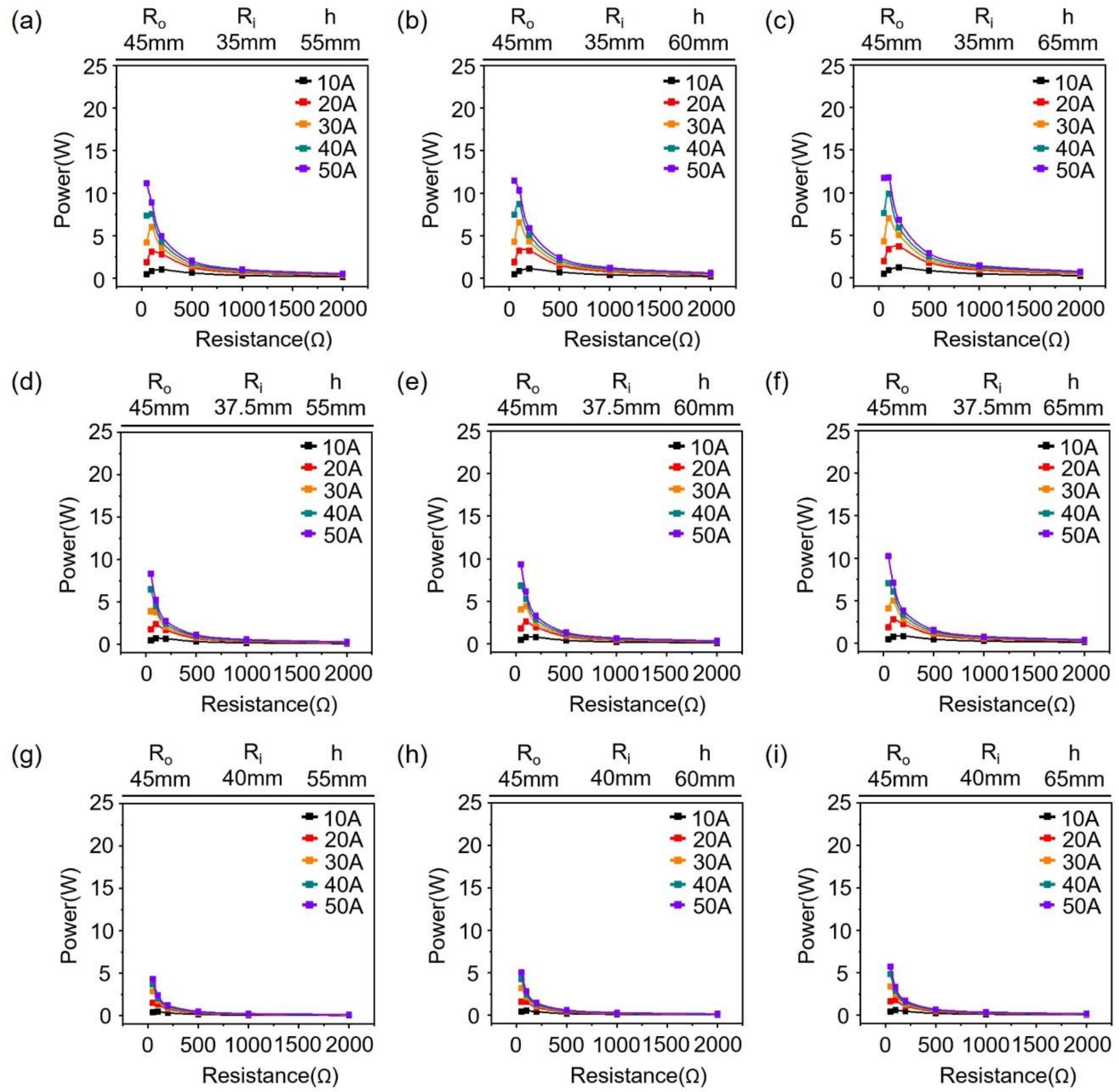

Figure. S2. Comparison of power vs resistance performance of various core geometry with R_i of 35mm, 37.5mm, and 40mm, h of 55mm, 60mm, and 65mm in simulation for the R_o of (a) to (i) 45mm respectively.

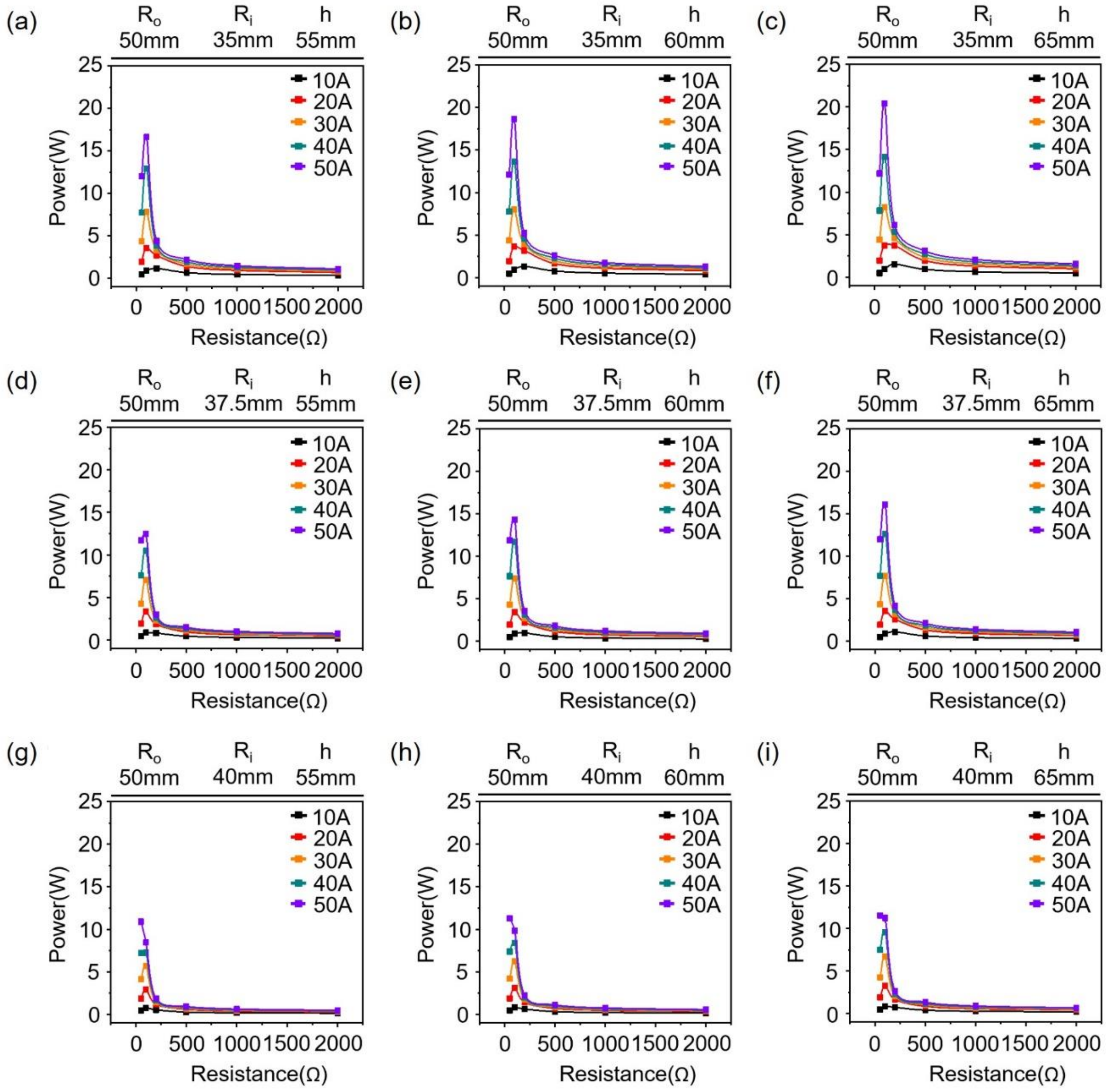

Figure S3. Comparison of power vs resistance performance of various core geometry with R_i of 35mm, 37.5mm, and 40mm, h of 55mm, 60mm, and 65mm in simulation for the R_o of (a) to (i) 50mm respectively.

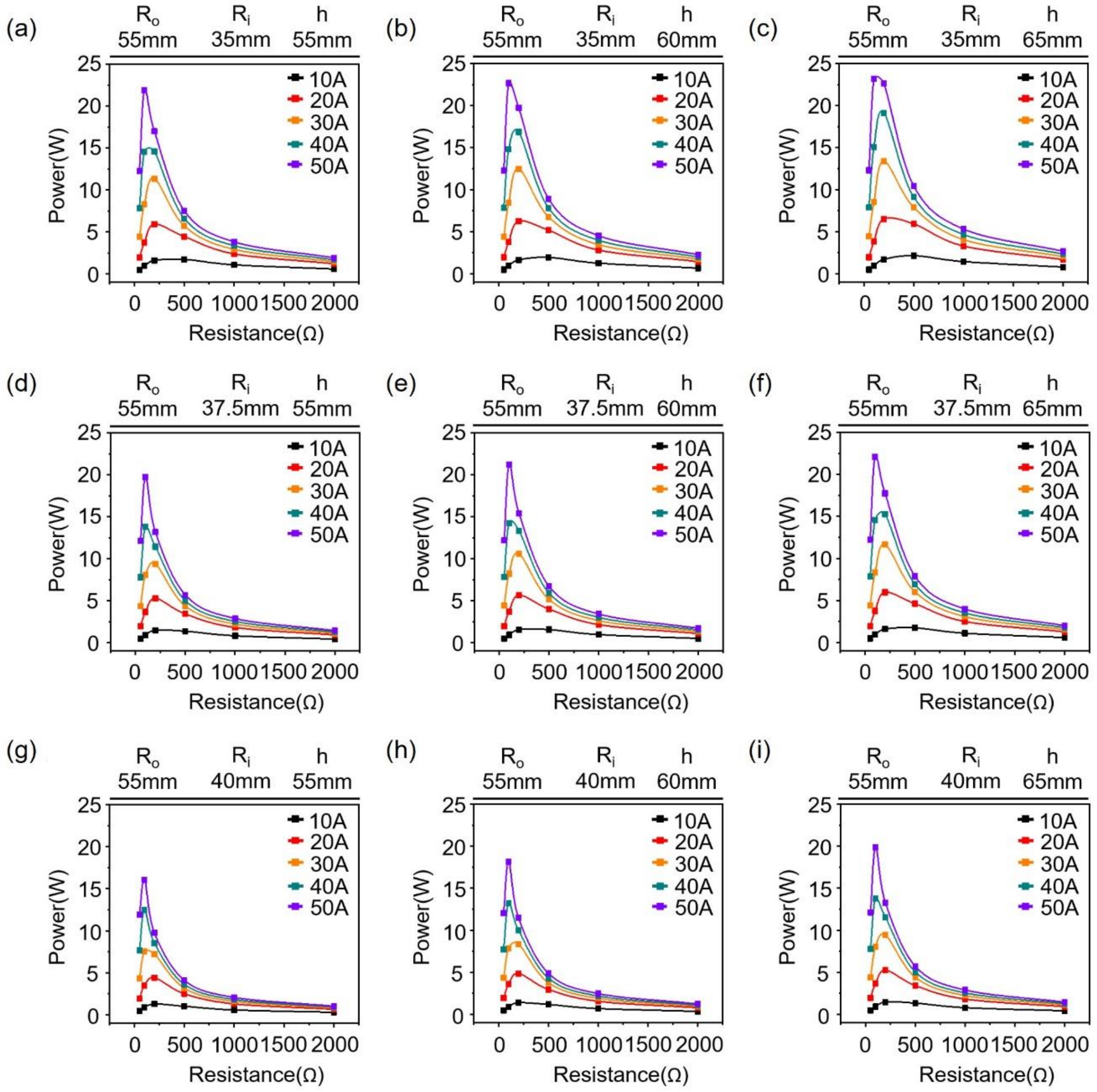

Figure S4. Comparison of power vs resistance performance of various core geometry with R_i of 35mm, 37.5mm, and 40mm, h of 55mm, 60mm, and 65mm in simulation for the R_o of (a) to (i) 55mm respectively.

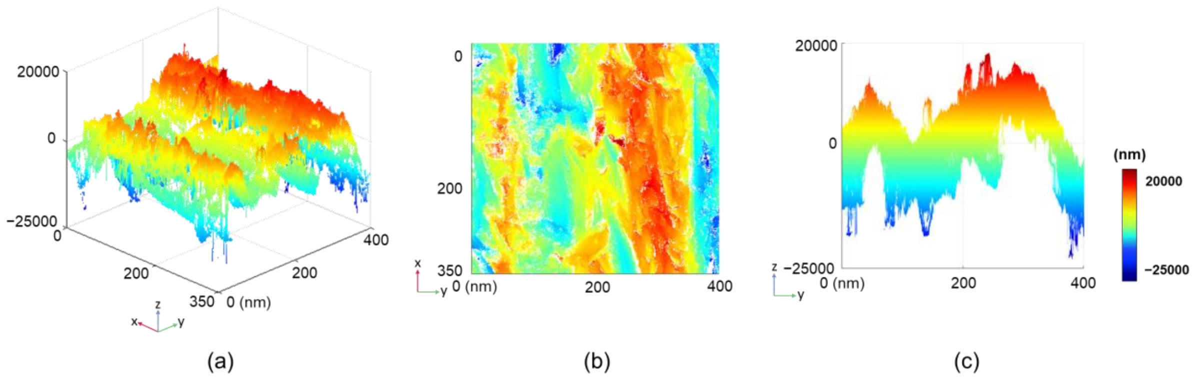

Figure S5. Surface roughness distribution before polishing process. (a) 3D surface roughness distribution of the corresponding cross-section. (b) Top view of the surface roughness distribution. (c) Side view of the surface roughness distribution with color bar indicating the roughness range from -25000 nm to 20000 nm.
